# A professional assessment of training plans for muscle hypertrophy and maximal strength developed by generative artificial intelligence

**DOI:** 10.5114/biolsport.2026.152350

**Published:** 2025-08-26

**Authors:** Tim Havers, Caroline Jelonnek, Lukas Masur, Eduard Isenmann, Billy Sperlich, Stephan Geisler, Peter Düking

**Affiliations:** 1 Department of Fitness and Health, IST University of Applied Sciences, Düsseldorf, Germany; 2 Department of Sport and Health Sciences, Technical University of Munich, Munich, Germany; 3 Department of Sports Science and Movement Pedagogy, Technische Universität Braunschweig, Braunschweig, Germany; 4 Integrative and Experimental Exercise Science and Training, Institute of Sport Science, University of Würzburg, Germany

**Keywords:** Resistance training, Artificial intelligence, Muscle hypertrophy, Maximal strength, Training plan design, Coaching evaluation, Chat bots, Large language models, Generative AI

## Abstract

The aim of this study was to evaluate the quality of resistance training plans for muscle hypertrophy and maximal strength generated by three large language models (LLMs): GPT-3.5 (via ChatGPT and Microsoft Copilot) and Google Gemini (GG). A total of 10 experienced coaches, each with at least a bachelor’s degree in exercise science and at least 2 years of coaching experience, rated these plans on a 1–5 Likert scale based on 27 criteria essential for effective training plan design. The LLMs were accessed on April 30, 2024, with a prompt structure that included key training objectives and the training history of a fictional advanced trainee. Results showed that the overall quality of the LLM-generated training plans was moderate. GG outperformed GPT-3.5 (via ChatGPT and Microsoft Copilot) for hypertrophy-related plans on 2 out of 27 criteria (advanced exercise methods, recovery strategies; p < 0.05), while GPT-3.5 (via Microsoft Copilot) outperformed GG for strength-related plans on 1 out of 27 criteria (testing procedure; p < 0.05). Across all criteria, GG received ratings > 3 more frequently than GPT-3.5 (via ChatGPT and Microsoft Copilot), particularly for general aspects, training principles, and training methods. Differences between hypertrophy- and strength-oriented plans within each LLM were minimal, although GPT-3.5 (via ChatGPT) showed the most inconsistency in ratings. Although LLM-generated plans can serve as an initial framework for hypertrophy and strength development, expert supervision remains crucial to refine these plans, as LLMs cannot account for individual responses to training, safety considerations, and the complex physiological adaptation processes observed by experienced coaches.

## INTRODUCTION

Resistance training is a fundamental component of physical activity that encompasses a broad range of goals, including e.g. muscle hypertrophy, strength development, injury prevention, rehabilitation, and overall health improvement. Resistance training benefits not only athletes across various sports but also recreationally active individuals seeking enhanced physical fitness and well-being [1–3]. Developing effective training programs, however, requires expertise in diverse fields such as exercise science [4], exercise biomechanics [5], and physiology [6, 7] to reduce the risk of suboptimal progress, inefficient training, or even health-related issues such as overtraining or injury. Certified trainers, coaches, and sports scientists typically acquire this specialized knowledge through dedicated education and years of practical experience. However, applying that knowledge in practice when developing training programs is time consuming. Publicly available Large Language Models (LLMs) are a promising alternative for creating individualised training plans, if used correctly. These include GPT 3.5 (OpenAI, San Francisco, USA) or Google Gemini (Alphabet Inc, Mountain View, USA) [8–12]. Although LLMs were not widely available during the COVID-19 pandemic, this period highlighted the need for accessible, remote training guidance, as many athletes relied on online resources and coaching by correspondence [13]. Despite their potential, the application of LLMs in exercise science and health promotion has been little explored.

Early research into ChatGPT and more contemporary models released by OpenAI has identified limitations in the individualization and overall quality of its exercise and health promotion plans [11, 14–17]. For example, Dergaa et al. examined GPT-4’s ability to prescribe exercise programs for five hypothetical cases and found that the generated plans often lacked precision in addressing individual health conditions and goals, prioritizing excessive safety over training effectiveness [14]. In a separate study, Dergaa et al. evaluated GPT-4’s capacity to generate personalized dietary advice for individuals with specific health conditions [17]. The study presented three hypothetical scenarios with distinct dietary requirements, which were assessed by a multidisciplinary team of nutritionists and physicians. While GPT-4 could generate meal plans aligned with general nutrition principles, the study highlighted limitations in addressing individual health factors, drug interactions, and setting realistic goals [17].

Düking et al. analyzed running training plans created by ChatGPT (version 3.0.1) based on prompts with varying levels of detail [15]. They concluded that even the best-performing plans contained suboptimal recommendations and lacked evidence-based prescriptions, though their quality improved with more detailed prompt inputs [15]. Similarly, Washif et al. evaluated resistance training plans for intermediate and advanced athletes generated by GPT-3.5 and GPT-4 via ChatGPT. They found that while the models could generate general plans, significant modifications were necessary to tailor them to specific training needs [11].

While some initial studies have examined the use of LLMs like ChatGPT in exercise science, research on other models, such as Google Gemini or models accessed via Microsoft Copilot, remains particularly scarce [18, 19]. Rocha-Silva assessed the similarity, readability, and quality of information provided by ChatGPT (3.5 and 4), Google Gemini, and Microsoft Copilot in response to questions about epilepsy and physical exercise [19]. The study found comparable response patterns across the models, ranging from good to acceptable, with ChatGPT-4 providing the highest quality responses according to expert evaluations [19]. This aligns with our previous research, which found generally suboptimal resistance training plans for hypertrophy generated by Google Gemini and GPT-4 via Microsoft Copilot. [18]. However, GPT-4 scored higher than Gemini on training plan ratings by experienced coaching professionals, defined as individuals with at least a bachelor’s degree in exercise science and at least three years of coaching experience in strength and conditioning or resistance training [18].

In designing training programs, the ability to differentiate training plans according to different goals is crucial. However, existing evidence suggests that LLMs, including GPT-3.5, GPT-4 (via ChatGPT and Microsoft Copilot), and Google Gemini, struggle with this differentiation [11, 18]. Washif et al. found that resistance training plans intended for maximal strength development often incorporated hypertrophy-focused elements, indicating a lack of specificity in addressing the primary objective [11]. Similarly, Havers et al. analyzed training plans generated by Google Gemini and GPT-4 (via Microsoft Copilot) and observed that hypertrophy-focused programs frequently included strength-oriented training components [18]. While muscle hypertrophy contributes to long-term strength development [3], training programs with time constraints should prioritize the most relevant goal to optimize results.

This challenge highlights the importance of training quality, a multifaceted concept that determines the effectiveness of a training program. Training quality can be assessed from two perspectives: 1) the quality of the overall training process, which includes goal setting, application of training principles, and long-term athlete development, and 2) the quality of specific training sessions, referring to the precision and execution of individual workouts in relation to their intended purpose [20]. In the context of this study, training quality refers specifically to the first dimension—how well resistance training plans generated by LLMs adhere to evidence-based principles and their suitability for specific training objectives.

Taken together, it remains unclear how effectively LLMs can distinguish between related but distinct training goals, such as muscle hypertrophy versus maximal strength development. This study seeks to address this gap with two primary objectives: (i) to evaluate the quality of resistance training plans focusing on muscle hypertrophy or maximal strength generated by publicly available LLMs (GPT-3.5 accessed via ChatGPT, GPT-3.5 accessed via Microsoft Copilot Microsoft, and Google Gemini) in terms of evidence-based criteria, and (ii) to compare the quality of training plans designed for muscle hypertrophy to those designed for maximal strength within each LLM.

## MATERIALS AND METHODS

### General Design

We addressed our first objective of analyzing resistance training plans by using different LLMs. We assessed the following LLMs: GPT-3.5 via ChatGPT, GPT-3.5 via Microsoft Copilot, and Google Gemini. We grounded our analytical approach in methodologies established in previous studies from the field of exercise science [15, 18]. We conducted the study through a structured approach: (i) applying criteria for evaluating resistance training plans based on recently published work [18], (ii) establishing standardized input information for the LLMs of interest, (iii) generating training plans focused on muscle hypertrophy and maximal strength development using defined input parameters, and (iv) engaging experienced coaching professionals to evaluate the generated plans based on the outlined criteria.

### Definition of criteria of relevance for resistance training-related training plans

Given the absence of a universally agreed-upon set of quality criteria for resistance training programs with specific objectives such as hypertrophy or maximal strength, we applied criteria derived from our recent work on muscle hypertrophy [15, 18]. The key aspects of designing effective training plans for hypertrophy and strength include the following:

1. Screening for individuals at increased risk of adverse exercise-related events, such as those associated with cardiovascular, pulmonary, metabolic, and other diseases [21].
2. Defining a clear training goal [22].
3. Establishing a reliable and valid testing procedure to assess initial performance status. This procedure should determine individual training variables (e.g., years of resistance training experience, body composition, prior training volume, and training weights) and define training outcomes (e.g., performance, physiological, subjective, biomechanical, or cognitive measures) [21–23].
4. Use training principles such as the principle of specificity (e.g. exercises tailored to a specific goal), the principle of progressive overload (e.g. gradual increases in intensity, load, repetitions or volume), the principle of variation (e.g. periodic adjustments in exercises, repetition ranges or intensities) and the principle of recovery (e.g. adequate rest between days or sessions targeting the same muscle group) to ensure training adaptations [22, 24].
5. Definition of basic strength training aspects including, but not limited to exercise selection, exercise order, and exercise technique (e.g., regarding safety aspects), as well as key variables like training frequency, intensity, and volume [3, 22, 25–27].

Beyond these general considerations, advanced aspects may also be incorporated into evidence-based training plans, including:

1. The use of advanced exercise methods like the manipulation of movement speed, range of motion or kinematics. Furthermore, time under tension can be manipulated as well as the set endpoint (e.g., ratings of perceived exertion [RPE], reps in reserve [RIR], proximity to failure) [26–28].
2. The integration of advanced unconventional training methods (e.g., drop sets, rest-pause training, or pre-exhaustion strategies), or specialized equipment like blood flow restriction bands [22, 25].
3. The implementation of advanced recovery strategies, including heat therapy, cooling, and optimized sleep routines [22].
4. The consideration of nutritional factors, such as macronutrient and micronutrient intake and hydration [22, 29].

### Definition of information input into publicly available LLMs

We have chosen GPT-3.5 accessed via ChatGPT, GPT-3.5 accessed via Microsoft Copilot, and Google Gemini, and inserted prompts on April 30^th^ 2024. These LLMs and their access points were chosen due to their widespread use and accessibility for everyday users. Furthermore, a direct comparison of these LLMs had not been conducted in the field of resistance training research prior to this work. Each objective (muscle hypertrophy [Prompt 1] and maximal strength development [Prompt 2] was addressed with a comprehensive prompt.

– *Prompt 1*: *Please provide me with a resistance training plan to increase muscle hypertrophy over the next 16 weeks. I am a 25-year-old female and have been doing resistance training 4 times a week for the past 6 years. Previous resistance training sessions have lasted 60–90 minutes. I have access to free weights and machines, both of which I would like to use. I also have training equipment such as belts, straps, bandages that I can use, and I have a body composition scale for monitoring purposes. My body weight is 60 kg with 20% body fat. I am 165 cm tall. I would like to increase the frequency to 5 times a week. I want to increase my total muscle mass as much as possible, although I am at an advanced level. I would like to emphasise my legs and my buttocks and have a more curvy silhouette. My one-repetition maximums in the squat, hip thrust, and deadlift are 100 kg, 175 kg, and 100 kg respectively. Overall, I want to incorporate advanced training strategies such as drop sets because I enjoy them. I also want to focus on my nutrition and recovery for muscle hypertrophy. To give you an idea of how I used to train, here is my previous resistance training programme:*
– *Prompt 2*: *Please provide me with a resistance training plan to increase maximal strength over the next 16 weeks. I am a 25-yearold female and have been doing resistance training 4 times a week for the past 6 years. Previous resistance training sessions have lasted 60–90 minutes. I have access to free weights and machines, both of which I would like to use. I also have training equipment such as belts, straps, bandages that I can use, and I have a body composition scale for monitoring purposes. I would like to keep my training frequency at 4 times a week. I want to increase my maximal strength as much as possible, although I am at an advanced level. I want to increase my maximal strength in squat, deadlift, and bench press by 10%. My one-repetition maximums in the squat, deadlift, and bench press are 100 kg, 100 kg, and 60 kg, respectively. Overall, I want to incorporate advanced training strategies such as cluster training because I enjoy it. I also want to focus on my nutrition and recovery. To give you an idea of how I used to train, here is my previous resistance training programme:*

**Table 1:** Previous hypertrophy-oriented resistance training plan provided as prompt (used by GPT-3.5 via ChatGPT and Microsoft Copilot, and Google Gemini to generate a new training plan)

**Day 1 & Day 3: Lower body & core**				
Exercises	Sets	Repetitions	Intensity	Rest periods
Romanian Deadlifts	4	8	1–2 reps in reserve	3 min.
Squats	3	10–12	3 reps in reserve	90 s
Hip Thrusts	3	8	1–2 reps in reserve	120 s
Bulgarian Split Squats	2	6–8	3 reps in reserve	3 min.
Leg raises	2	15–20	1–2 reps in reserve	90 s

**Day 2 & Day 4: Upper body**				
Exercises	Sets	Repetitions	Intensity	Rest periods
Rows	4	8	3 reps in reserve	3 min.
Bench press	3	10	3 reps in reserve	3 min.
Pulldown	3	10–12	1–2 reps in reserve	3 min.
Lateral raises	2	15–20	1–2 reps in reserve	90 s
Biceps curls	3	15	1–2 reps in reserve	90 s
Triceps ext.	3	15	1–2 reps in reserve	90 s

**Table 2:** Previous strength-oriented resistance training plan provided as second prompt (used by GPT-3.5 via ChatGPT and Microsoft Copilot, and Google Gemini to generate a new training plan)

**Day 1 & Day 3: Lower body & core**				
Exercises	Sets	Repetitions	Intensity	Rest periods
Deadlifts	5	1–3	0–2 reps in reserve	3–5 min.
Squats	5	1–3	0–2 reps in reserve	3–5 min.
Hip Thrusts	2	10–12	3 reps in reserve	120 s
Bulgarian Split Squats	2	10–12	3 reps in reserve	120 s
Leg raises	2	15–20	1–2 reps in reserve	90 s

**Day 2 & Day 4: Upper body**				
Exercises	Sets	Repetitions	Intensity	Rest periods
Bench press	5	1–3	0–2 reps in reserve	3–5 min.
Rows	4	8	1–2 reps in reserve	3 min.
Pulldown	3	10–12	3 reps in reserve	90 s
Lateral raises	2	15	1–2 reps in reserve	60 s
Biceps curls	2	15	1–2 reps in reserve	60 s
Triceps ext.	2	15	1–2 reps in reserve	60 s

The prompts were entered into each LLM of interest on the same day, April 30^th^, 2024, during the morning hours (between 9:00 and 10:00 AM), generating a total of six weekly training plans: one hypertrophy-related and one strength-related plan generated by GPT-3.5 via ChatGPT, as well as corresponding plans generated by GPT-3.5 via Microsoft Copilot and Google Gemini. All interactions were conducted on the same computer using unused accounts for each LLM to ensure consistency and control for potential updates. The full conversations with the LLMs are provided in the Appendix (S-Tables 1–6).

### Coaching professionals

The evaluation of LLM-derived training plans was conducted following the procedures outlined in previously published studies [15, 18, 30–32]. Experienced coaches assessed the resistance training plans, focusing on key aspects critical to effective training plan design, as detailed in Table 3. Each aspect was rated using a 1–5 Likert scale. To participate in the evaluation, coaches were required to hold at least a bachelor’s degree in exercise science and have a minimum of two years of direct, hands-on coaching experience in resistance training. Research suggests that fundamental principles of exercise prescription are largely consistent among trainers with comparable educational backgrounds, particularly those with an undergraduate degree in sport science [33]. This additional practical expertise was crucial for assessing the specificity and quality of the generated training plans.

**TABLE 3:** Relevant aspects when designing a training plan and the corresponding rating scale which was used to evaluate the training plans generated by Google Gemini and GPT-3.5 (accessed via ChatGPT and Microsoft Copilot).

Relevant aspects when designing a training plan		Rating (1–5 Likert Scale)						

		1 (bad)	2	3	4	5 (good)	Not Applicable	Comment
**General Aspects**	Overall training plan Health screening Defined goal Testing procedure regarding initial performance status Testing procedure regarding assessment of individual training variables Testing procedure regarding assessment of training effects Overall monitoring procedure							

**Training principles**	Principle of specificity Principle of progressive overload Principle of variation Principle of recovery							

**Basic strength training aspects**	Exercise selection Exercise order Weekly training frequency per muscle Training intensity per exercise Repetition range per exercise Overall training volume Number of sets per muscle per week Rest periods Exercise technique							

Advanced training aspects	Advanced exercise methods Time under tension Set endpoint Advanced training methods Equipment Recovery strategies Nutrition							

The study received approval from the Ethics Committee of the Faculty of Exercise Science and Training at the University of Würzburg (EV2023/7-2609) and was conducted in accordance with the Declaration of Helsinki.

### Statistics

We calculated descriptive statistics, including median and range, for the Likert scores across all 27 items. Given the ordinal nature of Likert scales, non-parametric tests were employed. Specifically, Friedman ANOVAs were used to analyze differences between strengthrelated and hypertrophy-related resistance training programs across the LLMs. When the Friedman ANOVA revealed significant results, pairwise comparisons were performed using Dunn’s test with Bonferroni correction. To determine whether there were significant differences between the ratings of hypertrophy and maximal strength programs within each LLM, we applied the Wilcoxon signed-rank test. The significance level for all tests was set at p < 0.05. Additionally, inter-rater reliability was assessed using Fleiss’ Kappa [34]. All statistical analyses were conducted in R (version 4.4.2) [35].

## RESULTS

A total of 10 experienced coaching professionals participated in this study (mean age: 30.2 ± 7.7 years; resistance training experience: 9.6 ± 5.1 years; recreational fitness coaching experience: 7.2 ± 6.1 years). Of these, nine held a Bachelor’s degree and one held a Diploma degree in Exercise Science. Each coach holds multiple certifications from both German and international training organizations (e.g., personal trainer certification, CrossFit certification, weightlifting coaching certification).

Fleiss’ Kappa values for the strength-oriented training plans generated by GPT-3.5 (via ChatGPT), Google Gemini, and GPT-3.5 (via Microsoft Copilot) were -0.007, -0.028, and 0.001 (overall: low agreement), respectively. For the hypertrophyoriented training plans, the Fleiss’ Kappa values were -0.006, -0.015, and -0.051 (overall: low agreement), respectively. All pvalues were > 0.05, indicating no significant inter-rater agreement. Likert scale distributions for each training plan are illustrated in the supplementary material (S-Figures 1–6).

### Differences between GPT-3.5 (via ChatGPT and via Microsoft Copilot) and Google Gemini

Descriptive statistics and results of significance testing between GPT3.5 (ChatGPT, Microsoft Copilot), and Google Gemini are presented in Table 4 (hypertrophy) and Table 5 (strength).

**TABLE 4:** Descriptive analysis (median and range) and results of significance tests of hypertrophy-oriented training programmes derived across LLMs (Google Gemini and GPT-3.5 accessed via ChatGPT and Microsoft Copilot)

Relevant aspects when designing a training plan		Median (Range)			Significance tests of hypertrophy-oriented training programmes derived across LLMs			

		ChatGPT	Gemini	Copilot	Friedmann ANOVA (p-value)	ChatGPT vs. Gemini (p-value)	ChatGPT vs. Copilot (p-value)	Gemini vs. Copilot (p-value)
**General Aspects**	Overall training plan	3.5 (2)	4 (2)	3 (3)	0.241	/	/	/
	Health screening	3 (3)	2 (4)	3 (4)	0.957	/	/	/
	Defined goal	3 (3)	3.5 (5)	3.5 (3)	0.226	/	/	/
	Testing procedure regarding initial performance status	2.5 (4)	3 (4)	2.5 (4)	0.638	/	/	/
	Testing procedure regarding assessment of individual training variables	2.5 (4)	4 (3)	3 (5)	**0.043**	0.096	0.934	0.775
	Testing procedure regarding assessment of training effects	3 (4)	3.5 (4)	2.5 (4)	0.141	/	/	/
	Overall monitoring procedure	2.5 (4)	3 (4)	2.5 (4)	0.727	/	/	/

**Summary Rating**	**< 3**	**n= 3**	**n = 1**	**n = 3**				
	**3**	**n = 3**	**n = 2**	**n = 3**				
	**> 3**	**n = 1**	**n = 4**	**n = 1**				

**Training principles**	Principle of specificity	3 (2)	4 (5)	4 (3)	0.284	/	/	/
	Principle of progressive overload	3 (3)	4 (5)	4 (3)	0.434	/	/	/
	Principle of variation	3 (5)	4 (5)	3.5 (3)	0.593	/	/	/
	Principle of recovery	4 (3)	5 (4)	4 (3)	0.179	/	/	/

**Summary Rating**	**< 3**	**n = 0**	**n = 0**	**n = 0**				
	**3**	**n = 3**	**n = 0**	**n = 0**				
	**> 3**	**n = 1**	**n = 4**	**n = 4**				

**Basic strength training aspects**	Exercise selection	4 (2)	4 (2)	4 (4)	0.303	/	/	/
	Exercise order	3 (3)	4 (3)	4 (2)	0.059	/	/	/
	Weekly training frequency per muscle	3.5 (2)	3.5 (3)	4 (3)	0.616	/	/	/
	Training intensity per exercise	4 (4)	4 (5)	3 (3)	0.166	/	/	/
	Repetition range per exercise	4 (3)	3.5 (3)	3 (3)	0.381	/	/	/
	Overall training volume	4 (4)	4.5 (2)	4 (3)	0.177	/	/	/
	Number of sets per muscle per week	3.5 (2)	3 (3)	4 (3)	0.695	/	/	/
	Rest periods	3.5 (5)	4 (3)	4 (5)	0.254	/	/	/
	Exercise technique	3 (4)	3.5 (5)	2 (4)	0.0914	/	/	/

**Summary Rating**	**< 3**	**n = 0**	**n = 0**	**n = 1**				
	**3**	**n = 2**	**n = 1**	**n = 2**				
	**> 3**	**n = 7**	**n = 8**	**n = 6**				

**Advanced training aspects**	Advanced exercise methods	2.5 (4)	4 (2)	3 (4)	**0.005**	**0.006**	0.802	0.143
	Time under tension	3 (4)	4 (3)	3.5 (5)	0.078	/	/	/
	Set endpoint	1.5 (4)	3 (4)	2.5 (5)	**0.016**	0.234	0.341	1.000
	Advanced training methods	3 (3)	3 (5)	2 (4)	0.405	/	/	/
	Equipment	3 (4)	3 (4)	2 (4)	0.180	/	/	/
	Recovery strategies	3 (3)	4 (4)	2 (4)	**0.008**	0.750	0.247	**0.011**
	Nutrition	3 (3)	2.5 (5)	2 (4)	0.45	/	/	/

**Summary Rating**	**< 3**	**n = 2**	**n = 1**	**n = 5**				
	**3**	**n = 5**	**n = 3**	**n = 1**				
	**> 3**	**n = 0**	**n = 3**	**n = 1**				

**TABLE 5:** Descriptive analysis (median and range) and results of significance tests of strength-oriented training programmes derived across LLMs (Google Gemini and GPT-3.5 accessed via ChatGPT and Microsoft Copilot)

Relevant aspects when designing a training plan		Median (Range)			Significance tests of strength-oriented training programmes derived across LLMs			

		ChatGPT	Gemini	Copilot	Friedmann ANOVA (p-value)	ChatGPT vs. Gemini (p-value)	ChatGPT vs. Copilot (p-value)	Gemini vs. Copilot (p-value)
**General Aspects**	Overall training plan	3 (5)	4 (3)	3 (2)	0.091	/	/	/
	Health screening	3 (4)	4 (4)	2.5 (4)	0.241	/	/	/
	Defined goal	3 (5)	4 (3)	3.5 (5)	0.727	/	/	/
	Testing procedure regarding initial performance status	3 (4)	4 (3)	2.5 (4)	0.067	/	/	/
	Testing procedure regarding assessment of individual training variables	3 (4)	4.5 (3)	2 (4)	**0.043**	0.159	1.000	**0.033**
	Testing procedure regarding assessment of training effects	2 (4)	3 (5)	3 (4)	0.289	/	/	/
	Overall monitoring procedure	3.5 (4)	3 (3)	3 (4)	0.227	/	/	/

**Summary Rating**	**< 3**	**n = 1**	**n = 0**	**n = 3**				
	**3**	**n = 5**	**n = 2**	**n = 3**				
	**> 3**	**n = 1**	**n = 5**	**n = 2**				

**Training principles**	Principle of specificity	3 (5)	3 (3)	3 (3)	0.549	/	/	/
	Principle of progressive overload	3.5 (5)	4 (3)	4 (5)	0.325	/	/	/
	Principle of variation	3.5 (3)	4 (4)	3 (4)	0.368	/	/	/
	Principle of recovery	3.5 (5)	3 (5)	3 (5)	0.156	/	/	/

**Summary Rating**	**< 3**	**n = 0**	**n = 0**	**n = 0**				
	**3**	**n = 1**	**n = 2**	**n = 3**				
	**> 3**	**n = 3**	**n = 2**	**n = 1**				

**Basic strength training aspects**	Exercise selection	4 (1)	4.5 (3)	4 (4)	0.301	/	/	/
	Exercise order	3 (3)	4 (3)	2.5 (3)	0.185	/	/	/
	Weekly training frequency per muscle	4 (2)	4.5 (3)	3 (3)	0.091	/	/	/
	Training intensity per exercise	4 (5)	4 (3)	4 (3)	0.852	/	/	/
	Repetition range per exercise	4 (3)	4 (4)	4 (3)	0.905	/	/	/
	Overall training volume	4 (2)	4 (4)	4 (3)	0.687	/	/	/
	Number of sets per muscle per week	4 (2)	4.5 (4)	3 (3)	0.055	/	/	/
	Rest periods	3 (5)	4 (4)	3 (3)	0.459	/	/	/
	Exercise technique	3 (4)	3 (4)	2 (4)	0.059	/	/	/

**Summary Rating**	**< 3**	**n = 0**	**n = 0**	**n = 2**				
	**3**	**n = 3**	**n = 1**	**n = 3**				
	**> 3**	**n = 6**	**n = 8**	**n = 4**				

**Advanced training aspects**	Advanced exercise methods	3 (4)	3 (5)	3 (5)	0.871	/	/	/
	Time under tension	4 (4)	4 (5)	4 (4)	0.309	/	/	/
	Set endpoint	3.5 (4)	3.5 (5)	4 (4)	0.638	/	/	/
	Advanced training methods	3 (3)	3 (5)	2.5 (5)	0.393	/	/	/
	Equipment	3 (5)	2.5 (5)	3 (4)	0.756	/	/	/
	Recovery strategies	3.5 (2)	4 (4)	4 (4)	0.481	/	/	/
	Nutrition	3.5 (3)	4 (4)	3 (2)	0.747	/	/	/

**Summary Rating**	**< 3**	**n = 0**	**n = 1**	**n = 1**				
	**3**	**n = 3**	**n = 2**	**n = 3**				
	**> 3**	**n = 4**	**n = 4**	**n = 3**				

### Differences in training quality between hypertrophy-oriented and strength-oriented training plans

Descriptive statistics and results of significance testing between hypertrophy-oriented and strength-oriented training plans within each LLM of interest are presented in Table 6.

**TABLE 6:** Descriptive analysis (median and range) and results of significance tests between strength and hypertrophy-oriented programmes

Relevant aspects when designing a training plan		Median (Range)						Significance tests between strength and hypertrophy-oriented programmes		

LLM of interest		ChatGPT		Gemini		Copilot		Wilcoxon Signed Rank Test (hypertrophy versus strength)		

Condition		Hypertrophy	Strength	Hypertrophy	Strength	Hypertrophy	Strength	ChatGPT (p-value)	Gemini (p-value)	Copilot
**General Aspects**	Overall training plan	3.5 (2)	3 (5)	4 (2)	4 (3)	3 (3)	3 (2)	1	1	0.41
	Health screening	3 (3)	3 (4)	2 (4)	4 (4)	3 (4)	2.5 (4)	1	0.139	0.824
	Defined goal	3 (3)	3 (5)	3.5 (5)	4 (3)	3.5 (3)	3.5 (5)	1	0.931	0.41
	Testing procedure regarding initial performance status	2.5 (4)	3 (4)	3 (4)	4 (3)	2.5 (4)	2.5 (4)	0.345	0.407	0.374
	Testing procedure regarding assessment of individual training variables	2.5 (4)	3 (4)	4 (3)	4.5 (3)	3 (5)	2 (4)	0.374	0.299	0.281
	Testing procedure regarding assessment of training effects	3 (4)	2 (4)	3.5 (4)	3 (5)	2.5 (4)	3 (4)	0.167	0.667	0.203
	Overall monitoring procedure	2.5 (4)	3.5 (4)	3 (4)	3 (3)	2.5 (4)	3 (4)	0.414	0.774	0.588

**Summary Rating**	**< 3**	**n = 3**	**n = 1**	**n = 1**	**n = 0**	**n = 3**	**n = 3**			
	**3**	**n = 3**	**n = 5**	**n = 2**	**n = 2**	**n = 3**	**n = 3**			
	**> 3**	**n = 1**	**n = 1**	**n = 4**	**n = 5**	**n = 1**	**n = 1**			

**Training principles**	Principle of specificity	3 (2)	3 (5)	4 (5)	3 (3)	4 (3)	3 (3)	1	0.586	0.167
	Principle of progressive overload	3 (3)	3.5 (5)	4 (5)	4 (3)	4 (3)	4 (5)	1	0.783	0.850
	Principle of variation	3 (5)	3.5 (3)	4 (5)	4 (4)	3.5 (3)	3 (4)	0.265	1	0.299
	Principle of recovery	4 (3)	3.5 (5)	5 (4)	3 (5)	4 (3)	3 (5)	0.343	**0.018**	0.097

**Summary Rating**	**< 3**	**n = 0**	**n = 0**	**n = 0**	**n = 0**	**n = 0**	**n = 0**			
	**3**	**n = 3**	**n = 1**	**n = 0**	**n = 2**	**n = 0**	**n = 3**			
	**> 3**	**n = 1**	**n = 3**	**n = 4**	**n = 2**	**n = 4**	**n = 1**			

**Basic strength training aspects**	Exercise selection	4 (2)	4 (1)	4 (2)	4.5 (3)	4 (4)	4 (4)	0.424	1	0.424
	Exercise order	3 (3)	3 (3)	4 (3)	4 (3)	4 (2)	2.5 (3)	0.766	1	0.305
	Weekly training frequency per muscle	3.5 (2)	4 (2)	3.5 (3)	4.5 (3)	4 (3)	3 (3)	0.140	0.322	0.269
	Training intensity per exercise	4 (4)	4 (5)	4 (5)	4 (3)	3 (3)	4 (3)	0.414	0.792	0.053
	Repetition range per exercise	4 (3)	4 (3)	3.5 (3)	4 (4)	3 (3)	4 (3)	0.783	0.796	**0.033**
	Overall training volume	4 (4)	4 (2)	4.5 (2)	4 (4)	4 (3)	4 (3)	0.671	0.498	0.572
	Number of sets per muscle per week	3.5 (2)	4 (2)	3 (3)	4.5 (4)	4 (3)	3 (3)	0.197	0.429	0.203
	Rest periods	3.5 (5)	3 (5)	4 (3)	4 (4)	4 (5)	3 (3)	1	0.565	0.340
	Exercise technique	3 (4)	3 (4)	3.5 (5)	3 (4)	2 (4)	2 (4)	0.240	1	0.773

**Summary Rating**	**< 3**	**n = 0**	**n = 0**	**n = 0**	**n = 0**	**n = 1**	**n = 2**			
	**3**	**n = 2**	**n = 3**	**n = 1**	**n = 1**	**n = 2**	**n = 3**			
	**> 3**	**n = 7**	**n = 6**	**n = 8**	**n = 8**	**n = 6**	**n = 4**			

**Advanced training aspects**	Advanced exercise methods	2.5 (4)	3 (4)	4 (2)	3 (5)	3 (4)	3 (5)	**0.033**	0.495	0.684
	Time under tension	3 (4)	4 (4)	4 (3)	4 (5)	3.5 (5)	4 (4)	**0.021**	0.586	0.931
	Set endpoint	1.5 (4)	3.5 (4)	3 (4)	3.5 (5)	2.5 (5)	4 (4)	**0.021**	0.43	0.197
	Advanced training methods	3 (3)	3 (3)	3 (5)	3 (5)	2 (4)	2.5 (5)	0.571	1	0.931
	Equipment	3 (4)	3 (5)	3 (4)	2.5 (5)	2 (4)	3 (4)	0.783	0.783	0.484
	Recovery strategies	3 (3)	3.5 (2)	4 (4)	4 (4)	2 (4)	4 (4)	0.088	0.346	0.054
	Nutrition	3 (3)	3.5 (3)	2.5 (5)	4 (4)	2 (4)	3 (2)	0.482	0.242	0.572

**Summary Rating**	**< 3**	**n = 2**	**n = 0**	**n = 1**	**n = 1**	**n = 4**	**n = 1**			
	**3**	**n = 5**	**n = 3**	**n = 3**	**n = 2**	**n = 1**	**n = 3**			
	**> 3**	**n = 0**	**n = 4**	**n = 3**	**n = 4**	**n = 1**	**n = 3**			

## DISCUSSION

Our findings reveal that the overall ratings of both hypertrophy- and strength-oriented training plans were moderate, with most criteria receiving average ratings on the 1–5 Likert scale (1 = poor, 5 = good). Only five criteria (i.e., testing procedure regarding assessment of individual training variables, principle of recovery, exercise selection, weekly training frequency per muscle, overall training volume) achieved ratings of 4.5 or higher on a 5-point Likert scale, all derived from Google Gemini.

Significant differences between hypertrophy-oriented training plans were found in two out of 27 criteria (advanced training methods and recovery strategies), with Google Gemini outperforming GPT-3.5 (via ChatGPT and Microsoft Copilot). For strength-based plans, only one criterion (testing procedure) showed significant differences, again in favor of Google Gemini.

Comparisons of training objectives (hypertrophy vs. strength) within the LLM of interest indicated similar overall quality, with few specific differences. The strength plan derived from GPT-3.5 (accessed via Microsoft Copilot) was rated higher than the hypertrophy plan in terms of basic aspects of strength training (repetition range per exercise). Similarly, the strength plan generated by GPT-3.5 (accessed via ChatGPT) outperformed the hypertrophy plan in terms of advanced training methodologies (advanced exercise methods, time under tension, set endpoints). However, the hypertrophy plan in Google Gemini outperformed the strength derived plan (principle of recovery). No other criteria differed significantly.

### Differences in quality of LLMs

To our knowledge, the only study directly comparing LLMs from different companies in the context of resistance training was conducted by our working group [18], whereas other studies have primarily examined different versions of an LLM from the same company [11]. In our previously published article [18], we compared training plans for muscle hypertrophy generated by Google Gemini and GPT-4 (via Microsoft Copilot) using both minimal and detailed input. Building on our previous findings, the response of LLMs can vary depending on the specific prompt [18]. The focus was on individuals with a basic understanding of training principles but limited experience in designing training plans. This target group is likely to benefit from the structured guidance and recommendations provided by LLMs, as they possess foundational training knowledge but may struggle with practical applications when designing their own plans. In the current study, we focus on the use of LLMs by experienced coaches and thereby only included detailed prompts instead of minimal input. Each objective (muscle hypertrophy [Prompt 1] and maximal strength development [Prompt 2]) was addressed with a comprehensive prompt to ensure that the plans were both detailed and well-structured. Experienced coaching professionals consistently rated GPT-4’s training plans higher than those from Gemini, regardless of input specificity [18]. This contrasts with our current findings, in which Gemini outperformed GPT-3.5 (via ChatGPT) in one of 27 criteria for the hypertrophy plan and surpassed GPT-3.5 (via Microsoft Copilot) in one of 27 criteria for both hypertrophy and strength plans. The reasons for these discrepancies remain speculative but may be attributed to differences in the underlying architectures, training data, or reasoning capabilities of each LLM. However, since exact algorithms underlying the LLMs are not publicly available, we cannot be certain about these factors and their specific influence. For example, GPT-4 has demonstrated superior contextual reasoning and coding proficiency compared to its predecessor GPT-3.5 [11], which may explain why it received higher ratings in previous research. However, Gemini’s more recent model updates and different training methodologies could have influenced its relative performance against GPT-3.5 in the present study. Washif et al. evaluated GPT-3.5 and GPT-4 (both accessed via ChatGPT) for resistance training programming and found that, while both models often lacked sufficient detail (e.g., exercise selection, tempo), GPT-4 generally outperformed GPT-3.5 [11]. Similarly, Puce et al. assessed the sports nutrition knowledge of GPT-4 and GPT-3.5 (both accessed via ChatGPT), finding greater accuracy in GPT-4 [36]. Further research is needed to systematically investigate how reasoning ability, data sources, and architecture contribute to differences in LLM performance for exercise programming.

Comparing the performance of publicly available LLMs in other exercise- or health-related settings has revealed no consistent pattern of superiority [17, 36, 37]. Concerning nutritional knowledge, Puce et al. compared the versions of GPT-3.5 accessed via Microsoft Copilot, Google Bard (which transitioned to Google Gemini on February 8, 2024; [9]) and GPT-3.5 accessed via ChatGPT and reported the highest accuracy for GPT-3.5 accessed via Microsoft Copilot (92%), followed by Google Bard (84%), and GPT-3.5 via ChatGPT [36]. Similarly, Dergaa et al. evaluated ChatGPT’s ability to provide tailored nutrition advice and found that while it could generate meal plans aligned with basic nutritional principles, it lacked clinical reasoning, particularly in handling health conditions and drug interactions [17]. The authors emphasized the need for human oversight and interdisciplinary collaboration to refine AI-generated dietary plans before practical application [17]. In contrast, Naz et al. compared Google Gemini, GPT 3.5 (via ChatGPT), and GPT-3.5 (via Microsoft Copilot) in the context of chronic kidney disease information and found that Google Gemini achieved the highest global quality scores, outperforming both GPT-3.5 models [37]. These findings further illustrate that the relative performance of LLMs can vary significantly depending on the specific application and evaluation criteria.

Overall, the hypertrophy- and strength-related training plans generated by GPT-3.5 (ChatGPT and Microsoft Copilot) and Google Gemini received similar ratings. Google Gemini outperformed GPT-3.5 (accessed via ChatGPT and accessed via Microsoft Copilot) in 2 out of 27 criteria for hypertrophy-related training plans and exceeded GPT-3.5 (via Microsoft Copilot) in 1 out of 27 criteria for strength-related plans. However, ratings rarely surpassed 4 on a 1–5 Likert scale, highlighting the need for fine-tuning by experienced practitioners. These findings align with previous research in exercise science [15, 18], medicine [38], and health promotion [14], which suggests that LLMs are most effective as tools for developing preliminary frameworks that require expert refinement to ensure evidencebased individualization.

### Differences between hypertrophy and strength training plans

By comparing hypertrophy- and strength-derived training plans within each LLM, we found that strength-focused plans received higher overall ratings in GPT-3.5 via ChatGPT (with 3 out of 27 items showing significant differences) and Microsoft Copilot (1 out of 27 items). In contrast, the hypertrophy-focused plan generated by Google Gemini was rated higher than the strength derived plan in 1 out of 27 items by experienced coaching professionals.

### GPT 3.5 accessed via ChatGPT

For ChatGPT, the criteria ‘advanced exercise methods’, ‘time under tension’ and ‘set endpoint’ were rated significantly higher in the strength-based training plan. Both derived resistance training plans included advanced training methods such as drop sets (in the hypertrophy plan) and cluster sets (in the strength plan), which have been shown to be effective in promoting neuromuscular adaptations [39, 40].

The strength-oriented plan provided more detailed instructions on cluster sets, which probably contributed to its higher ratings by the experienced coaching professionals. While both plans from GPT-3.5 (accessed via ChatGPT) lacked comprehensive information on key parameters such as ‘time under tension’ and ‘set endpoints’ (e.g. proximity to muscle failure), the strength-oriented plan provided some detail on time under tension for specific exercises such as the weighted plank, a feature missing from the hypertrophy plan. Neither plan explicitly addressed proximity to failure, but the strengthoriented plan included intensity descriptions, such as percentage of one repetition maximum, but only during the power phase for exercises such as speed bench press. Moreover, the hypertrophy-oriented training plan provided structured set and repetition schemes but lacked explicit intensity recommendations. Instead, it included general guidelines such as: “progressively overload your muscles by increasing weights, reps, or sets every 1–2 weeks to continue stimulating muscle growth” and “listen to your body and adjust intensity as needed to avoid overtraining and injury” (see supplementary material). Notably, such guidance was not incorporated in the strengthoriented plan.

From a training effectiveness perspective, providing recommendations for progressive overload is essential because it ensures continuous adaptation to training stimuli [24]. However, in the absence of precise intensity guidelines, trainees may unintentionally train at suboptimal intensities that either exceed or fall short of the stimulus required for effective adaptation [41]. This issue is particularly relevant given that intensity regulation is a key determinant of hypertrophy and strength development [4]. In contrast, real-world coaching practices typically include explicit intensity prescriptions, such as percentage of one repetition maximum (%1-RM), ratings of perceived exertion (RPE), or repetitions in reserve (RIR), to optimize training progression.

Furthermore, the recommendations in the hypertrophy plan adopt a broad and conservative approach, likely prioritizing safety over optimal training effectiveness [14]. While injury prevention is a crucial aspect of resistance training, excessive caution may result in suboptimal training stimuli, particularly for well-trained individuals who require more precise intensity guidelines to sustain progress. This aligns with the findings of Dergaa et al., who noted that AI-generated training plans, despite their structured nature, often lack the intensity regulation and variability necessary for meaningful long-term improvements in athletic performance [14].

A key limitation of LLM-generated programs is that, while these models possess extensive domain knowledge, they lack the ability to engage in clinical reasoning. This gap prevents them from dynamically integrating critical training principles, such as individualized intensity prescriptions, into program design [11, 38]. Dergaa et al. highlighted this limitation in a psychiatric context, showing that while ChatGPT provided appropriate advice for straightforward cases, its effectiveness deteriorated in complex scenarios requiring nuanced decision-making [38]. Similarly, Washif et al. observed that GPT-3.5- and GPT-4-generated resistance training plans failed to incorporate advanced methods known to enhance strength adaptations, such as cluster sets, variable resistance training, and blood flow restriction. Additionally, time-efficient and effective techniques like supersets and drop sets were omitted, suggesting a misalignment with contemporary, evidence-based training methodologies. Interestingly, our analysis found that the LLM-generated training plans did include drop sets (hypertrophy plan) and cluster sets (strength plan) (see supplementary material). However, this inclusion was likely a direct result of the specific prompt instructions rather than an autonomous decision by the model. This suggests that LLMs may struggle to independently propose advanced training strategies unless explicitly directed to do so, reinforcing their reliance on user input rather than adaptive, context-aware reasoning.

These findings highlight the importance of adequately addressing key training parameters, such as time under tension and training intensity (e.g., proximity to failure), in resistance training plans. Appropriate training modulation of these parameters is crucial as they significantly influence training adaptations [26, 28, 42].

### GPT 3.5 accessed via Microsoft Copilot

For Microsoft Copilot, the only criterion that was rated significantly differently was ‘repetition range per exercise,’ which was rated higher in the strength-oriented plan (median: 4) compared to the hypertrophyoriented plan (median: 3). The strength plan included a twice-weekly training frequency for the squat, bench press, and deadlift, with repetition ranges of 1–3 for these priority exercises, whereas higher repetition ranges (8–15) were assigned to non-priority exercises. In addition, the plan specified a proximity to failure of 0–3 repetitions in reserve, reflecting a training intensity sufficient to induce neuromuscular adaptations for maximal strength development [42–44]. In contrast, the hypertrophy plan proposed 4–5 sets of 8–12 repetitions per muscle group per session. Whilst this repetition range and volume are well-suited to stimulate muscular hypertrophy [3, 25], the plan lacked critical information about training intensity, such as proximity to failure or the percentage of one-repetition maximum, similar to GPT-3.5 accessed via ChatGPT. This omission raises concerns about whether the plan implicitly assumes generalized muscle failure as an endpoint, a potential shortcoming that may explain why the strengthoriented plan received higher ratings on this criterion.

As with the training plans generated by GPT-3.5, the Microsoft Copilot-derived programs followed a structurally sound framework but lacked the dynamic progression and individualization necessary for sustained training effectiveness. The unquestioned implementation of set and repetition recommendations could lead to an undershooting or overshooting of training loads over time, which in turn might either increase the risk of injury or result in inadequate adaptations, particularly for trained individuals [41]. As mentioned, the conservative nature of these plans suggests that LLMs prioritize safety over optimal training effectiveness. This cautious approach may stem from an inherent limitation in AI-generated programming, as models cannot take responsibility for training outcomes.

### Google Gemini

For Google Gemini, the item ‘principle of recovery’ received higher ratings in the hypertrophy plan compared to the strength plan. Although both plans emphasized the importance of ‘proper recovery, neither provided specific details on what should be recovered from or the underlying rationale.

The hypertrophy plan followed a five-day structure: day 1 (legs and glutes), day 2 (push: chest, shoulders, triceps), day 3 (rest/active recovery, e.g., cardio or yoga), day 4 (pull: back, biceps), and day 5 (lower body), with days 6 and 7 designated as rest days. This structure is partially aligned with the input prompt, which specified five weekly training sessions with a focus on the lower body. However, inconsistencies arose, particularly in counting day 3 (active recovery) as a full training session, reflecting a lack of precision in program design. Nonetheless, from a recovery standpoint, the frequency of two lower-body sessions per week appears sufficient for hypertrophic adaptations, provided the training volume is well distributed [3, 25, 45]. Specifically, the plan included 18 weekly direct sets for the quadriceps, supporting the idea that splitting volume across two sessions optimizes recovery and performance [3]. However, the hypertrophy plan also featured a strength-oriented day, incorporating back squats, deadlifts, and leg presses with 3–5 sets of 3–5 repetitions. While low-repetition training can still promote hypertrophy if total volume is sufficient [44], this approach raises concerns regarding goal alignment. Additionally, the higher intensities associated with such schemes may increase injury risk [46], highlighting the need for more precise programming.

The strength plan followed a four-day structure: day 1 (lower body and core), day 2 (upper body push), day 3 (rest/active recovery), and day 4 (upper body pull). Similar to the hypertrophy plan, it lacked a clear distinction between recovery and training days. The main lifts (squat, bench press, and deadlift) were trained only once per week, with 3–5 sets per lift, depending on the week. While indirect volume from assistance exercises likely contributed to adaptations [3, 47, 48], the low frequency of these key lifts could limit neuromuscular improvements, as multi-joint movements often benefit from higher training frequencies [49].

Ultimately, the hypertrophy plan received better ratings, likely due to its more balanced recovery distribution. While the strength plan’s frequency may have been sufficient for recovery, its limited exposure to the main lifts and lack of structured recovery strategies highlights areas for improvement.

### Strength, limitations and future research

A key strength of our study is that it represents the first direct comparison of resistance training plans for muscle hypertrophy and maximal strength development across LLMs from different companies. Our findings emphasize the need for practitioners to refine LLMgenerated training plans, reinforcing that these models cannot replace human expertise in coaching. However, several limitations must be acknowledged.

Overall plan scores rarely exceeded 4 on the Likert scale, with the only perfect rating awarded to Google Gemini’s hypertrophy plan for the criterion “principle of recovery”. This suggests that while LLM-generated plans provide a foundational framework, they are not without flaws. These findings align with previous research in exercise science [15, 18], medicine [38], and health promotion [14], which consistently suggests that LLMs serve best as preliminary drafting tools requiring expert refinement for evidencebased individualization.

Moreover, the use of a 5-point Likert scale provides only a first approach for assessing the quality of training plans. While it allows general trends and differences between LLMs to be identified, it may not fully capture the nuances of each program. Furthermore, it remains questionable whether a 5/5 rating can be achieved by either an LLM or a human coach.

The low Fleiss’ Kappa values observed in this study indicate low interrater reliability across all training plans, mirroring previous research [15, 18]. Although all participating raters had academic and practical coaching experience, differences in their familiarity with contemporary resistance training guidelines or personal preferences and experiences may have influenced their evaluations. This variability underscores the difficulty of reaching consensus in training plan assessments and suggests the need for more refined expert selection criteria. Different coaches may prioritize hypertrophy- and strength-related aspects differently, which should be taken into account in future studies [18].

Whilst our criteria for evaluating generated training plans are in line with previous research and training principles [15, 18], we acknowledge that they may not be of equal importance for all individuals. For example, for individuals who are relatively new to strength and/or hypertrophy training, the quality criteria ‘advanced exercise methods’ (e.g. manipulation of speed, range of motion of certain exercises), ‘set endpoint’ or ‘advanced training methods’ (e.g. drop sets) may be less important than for individuals who have years of strength and/or hypertrophy training experience and adaptations. Consequently, a limitation of our study is that we did not assess the relative importance of each quality criterion. It is possible that some criteria are more relevant to certain individuals than others.

It is important to acknowledge that our findings are based on the basic versions of large language models (GPT-3.5 accessed via ChatGPT, Microsoft Copilot; and Google Gemini) as of April 30, 2024. This represents a specific snapshot in LLM development, and given the rapid evolution of AI technologies, the long-term applicability of our results is inherently limited.

While GPT-4 has been shown to outperform GPT-3.5 in various contexts, including the generation of more accurate and detailed training plans [14, 19], our study was limited to GPT-3.5 (accessed via ChatGPT and Microsoft Copilot). Prior research, such as that by Rocha-Silva et al. [19], has highlighted the superior response quality of GPT-4 over GPT-3.5, and similar findings have been observed in resistance training plan generation, where GPT-4 outperformed GPT-3.5 and Google Gemini [14, 18]. Future iterations of LLMs may further refine training recommendations, potentially leading to more precise, goal-specific, and adaptable programs. This continuous advancement presents both a challenge and an opportunity. While it necessitates ongoing research to evaluate the effectiveness of newer models, it also opens the door for AI-generated training programs to become more refined, incorporating up-to-date sports science insights and personalized adaptations. Future research should explore these newer iterations, assessing their ability to generate evidencebased, individualized resistance training plans with improved accuracy, adaptability, and safety considerations.

## CONCLUSIONS

This study evaluated hypertrophy- and strength-focused resistance training plans generated by GPT-3.5 (via ChatGPT and Microsoft Copilot) and Google Gemini. The findings suggest that while LLMs can generate structured programs, their overall quality is moderate, with most plans scoring below 4 on a 1–5 Likert scale. Google Gemini slightly outperformed GPT-3.5 in a few isolated criteria, but no model consistently excelled across all parameters. Differences between hypertrophy- and strength plans within each LLM were minimal, with GPT-3.5 showing the most inconsistencies. A limitation across all models was the lack of detailed intensity parameters, such as proximity to failure and load prescription, which are crucial for effective training. LLMs tended to prioritize safety over optimal effectiveness, often providing conservative recommendations. Fitness professionals and recreational users should treat these plans as templates that require further adjustment based on individual needs and scientific principles. Future research should focus on evaluating other and contemporary LLM versions, such as GPT-4, and incorporating domain-specific training data to improve the quality and customization of resistance training programs.

## Supplementary Material

A professional assessment of training plans for muscle hypertrophy and maximal strength developed by generative artificial intelligence
