# Supplementary material for: A professional assessment of training plans for muscle hypertrophy and maximal strength developed by generative artificial intelligence
Source: Biol Sport. 2025 Aug 26;42(4):353–66. doi: 10.5114/biolsport.2026.152350 (PMC12492345; doi:10.5114/biolsport.2026.152350)
Supplement: A professional assessment of training plans for muscle hypertrophy and maximal strength developed by generative artificial intelligence [file JBS-42-4-56339-s1.pdf]

## SUPPLEMENTARY MATERIAL

**S-TABLE 1:** Conversation with GPT-3.5 (via ChatGPT; hypertrophy) on April 30, 2024

**Inserted Question by Human:**

Please provide me with a resistance training plan to increase muscle hypertrophy over the next 16 weeks. I am a 25-year-old female and have been doing resistance training 4 times a week for the past 6 years. Previous resistance training sessions have lasted 60–90 minutes. I have access to free weights and machines, both of which I would like to use. I also have training equipment such as belts, straps, bandages that I can use, and I have a body composition scale for monitoring purposes. My body weight is 60 kg with 20% body fat. I am 165 cm tall. I would like to increase the frequency to 5 times a week. I want to increase my total muscle mass as much as possible, although I am at an advanced level. I would like to emphasise my legs and my buttocks and have a more curvy silhouette. My one-repetition maximums in the squat, hip thrust, and deadlift are 100 kg, 175 kg, and 100 kg respectively. Overall, I want to incorporate advanced training strategies such as drop sets because I enjoy them. I also want to focus on my nutrition and recovery for muscle hypertrophy. To give you an idea of how I used to train, here is my previous resistance training programme:

| **Day 1 & Day 3: Lower body & core** | | | | |
|---|---|---|---|---|
| Exercises | Sets | Repetitions | Intensity | Rest periods |
| Romanian Deadlifts | 4 | 8 | 1–2 reps in reserve | 3 min. |
| Squats | 3 | 10–12 | 3 reps in reserve | 90 s |
| Hip Thrusts | 3 | 8 | 1–2 reps in reserve | 120 s |
| Bulgarian Split Squats | 2 | 6–8 | 3 reps in reserve | 3 min. |
| Leg raises | 2 | 15–20 | 1–2 reps in reserve | 90 s |
| **Day 2 & Day 4: Upper body** | | | | |
| Exercises | Sets | Repetitions | Intensity | Rest periods |
| Rows | 4 | 8 | 3 reps in reserve | 3 min. |
| Bench press | 3 | 10 | 3 reps in reserve | 3 min. |
| Pulldown | 3 | 10–12 | 1–2 reps in reserve | 3 min. |
| Lateral raises | 2 | 15–20 | 1–2 reps in reserve | 90 s |
| Biceps curls | 3 | 15 | 1–2 reps in reserve | 90 s |
| Triceps ext. | 3 | 15 | 1–2 reps in reserve | 90 s |

**Response Generated by ChatGPT:**

Given your experience level and goals, here's a 16-week resistance training plan that incorporates your preferences for advanced training strategies like drop sets, as well as a focus on nutrition and recovery for muscle hypertrophy:

- **Frequency:** 5 days per week
- **Training Split:**
  - Day 1: Lower Body (emphasis on legs and buttocks)
  - Day 2: Upper Body
  - Day 3: Lower Body (emphasis on legs and buttocks)
  - Day 4: Upper Body
  - Day 5: Lower Body (emphasis on legs and buttocks)
  - Day 6: Rest or Active Recovery
  - Day 7: Rest
- **Nutrition and Recovery:**
  - Ensure you're consuming enough protein to support muscle growth, aiming for around 1.6–2.2 grams per kilogram of body weight per day.
  - Prioritize post-workout nutrition with a combination of protein and carbohydrates to support muscle recovery and glycogen replenishment.
  - Incorporate rest days and active recovery activities like yoga, walking, or swimming to aid in muscle recovery and prevent overtraining.

  - Focus on getting adequate sleep (7–9 hours per night) to support muscle repair and growth.
  - Stay hydrated throughout the day, aiming for at least 3–4 liters of water daily.
- **Progression:**
  - Aim to progressively overload your muscles by increasing weights, reps, or sets every 1–2 weeks to continue stimulating muscle growth.
  - Listen to your body and adjust intensity and volume as needed to avoid overtraining and injury.

**Weeks 1–4: Hypertrophy Phase**

**Day 1: Lower Body** (emphasis on legs and buttocks)
**Exercise Sets Repetition Notes**
Romanian Deadlifts 4 8 –
Squats 3 10 –
Hip Thrusts 3 8 –
Bulgarian Split Squats 3 6–8 –
Leg Press 3 12 incorporate drop sets on the last set
Leg Curls 3 10–12 –
Calf Raises 3 15 –

**Day 2: Upper Body**
**Exercise Sets Repetition Notes**
Pull-Ups or
Lat Pulldowns 4 8 –
Bench Press 3 10 –
Rows 3 10 –
Shoulder Press 3 8 –
Dumbbell Flys 3 12 –
Biceps Curls 3 12 incorporate drop sets on the last set
Triceps Pushdowns 3 12 –

**Day 3: Lower Body** (emphasis on legs and buttocks)
**Exercise Sets Repetition Notes**
Squats 4 6 increased intensity
Deadlifts 3 8 –
Bulgarian Split Squats 3 8 –
Hip Thrusts 3 8 –
Walking Lunges 3 12 per leg
Leg Extensions 3 12 –
Seated Calf Raises 3 15 –

**Day 4: Upper Body**
**Exercise Sets Repetition Notes**
Bent Over Rows 4 8 –
Incline Bench Press 3 10 –
Pull-Ups or Lat Pulldowns 3 10 –
Lateral Raises 3 12 –
Barbell Curl 3 12 incorporate drop sets on the last set
Skull Crushers 3 12 –

**Day 5: Lower Body** (emphasis on legs and buttocks)
**Exercise Sets Repetition Notes**
Sumo Deadlifts 4 6 increased intensity
Front Squats 3 8 –
Romanian Deadlifts 3 8 –
Glute Bridges 3 10 incorporate drop sets on the last set
Leg Press 3 12 –
Standing Calf Raises 3 15 –

**S-TABLE 2:** Conversation with GPT-3.5 (via ChatGPT; strength) on April 30, 2024

**Inserted Question by Human:**

Please provide me with a resistance training plan to increase maximal strength over the next 16 weeks. I am a 25-year-old female and have been doing resistance training 4 times a week for the past 6 years. Previous resistance training sessions have lasted 60–90 minutes. I have access to free weights and machines, both of which I would like to use. I also have training equipment such as belts, straps, bandages that I can use, and I have a body composition scale for monitoring purposes. I would like to keep my training frequency at 4 times a week. I want to increase my maximal strength as much as possible, although I am at an advanced level. I want to increase my maximal strength in squat, deadlift, and bench press by 10%. My one-repetition maximums in the squat, deadlift, and bench press are 100 kg, 100 kg, and 60 kg, respectively. Overall, I want to incorporate advanced training strategies such as cluster training because I enjoy it. I also want to focus on my nutrition and recovery. To give you an idea of how I used to train, here is my previous resistance training programme:

| **Day 1 & Day 3: Lower body & core** | | | | |
|---|---|---|---|---|
| Exercises | Sets | Repetitions | Intensity | Rest periods |
| Deadlifts | 5 | 1–3 | 0–2 reps in reserve | 3–5 min. |
| Squats | 5 | 1–3 | 0–2 reps in reserve | 3–5 min. |
| Hip Thrusts | 2 | 10–12 | 3 reps in reserve | 120 s |
| Bulgarian Split Squats | 2 | 10–12 | 3 reps in reserve | 120 s |
| Leg raises | 2 | 15–20 | 1–2 reps in reserve | 90 s |

| **Day 2 & Day 4: Upper body** | | | | |
|---|---|---|---|---|
| Exercises | Sets | Repetitions | Intensity | Rest periods |
| Bench press | 5 | 1–3 | 0–2 reps in reserve | 3–5 min. |
| Rows | 4 | 8 | 1–2 reps in reserve | 3 min. |
| Pulldown | 3 | 10–12 | 3 reps in reserve | 90 s |
| Lateral raises | 2 | 15 | 1–2 reps in reserve | 60 s |
| Biceps curls | 2 | 15 | 1–2 reps in reserve | 60 s |
| Triceps ext. | 2 | 15 | 1–2 reps in reserve | 60 s |

**Response Generated by ChatGPT:**

Given your experience level and goals, here's a 16-week resistance training plan tailored to increase your maximal strength in squat, deadlift, and bench press by 10%. This plan incorporates advanced training strategies like cluster training and focuses on nutrition and recovery.
Remember to listen to your body and adjust the intensity and volume as needed. Consistency, proper nutrition, and adequate recovery are key to maximizing strength gains.

- **Frequency: 4 days per week**
- **Training Split:**
  - Day 1: Lower Body & Core
  - Day 2: Upper Body
  - Day 3: Rest or Active Recovery
  - Day 4: Lower Body & Core
  - Day 5: Upper Body
  - Day 6–7: Rest or Active Recovery
- **Nutrition and Recovery:**
  - Caloric Intake: Ensure you're in a slight caloric surplus to support muscle growth and strength gains.
  - Macronutrients: Aim for adequate protein intake (around 1.6–2.2 g/kg body weight), sufficient carbohydrates for energy, and healthy fats.
  - Hydration: Drink plenty of water to stay hydrated, especially during intense training sessions.
  - Sleep: Aim for 7–9 hours of quality sleep per night to support recovery and muscle growth.
  - Recovery Modalities: Incorporate foam rolling, stretching, and mobility work into your routine to enhance recovery and reduce the risk of injury.

**Week 1–4: Hypertrophy Phase**

**Day 1: Lower Body & Core**

**Exercise Sets Repetition Notes**

Deadlifts 4 3 Cluster: 3 sets of 3 reps with 20–30 sec rest in between clusters, 3–5 min rest between sets

Squats: 4 3 Cluster: 3 sets of 3 reps with 20–30 sec rest in between clusters, 3–5 min rest between sets

Bulgarian Split Squats 3 8 –

Hip Thrusts 3 10 –

Weighted Planks 3 45 s –

**Day 2: Upper Body**

**Exercise Sets Repetition Notes**

Bench Press 4 3 Cluster: 3 sets of 3 reps with 20–30 sec rest in between clusters, 3–5 min rest between sets

Rows 4 8 –

Pull-ups or Pulldowns 3 8–10 –

Dumbbell Shoulder Press 3 8 –

Biceps Curls 3 10 –

Triceps Dips 3 10 –

**Week 5–8: Strength Phase**

**Day 1: Lower Body & Core**

**Exercise Sets Repetition Notes**

Deadlifts 5 2 Cluster: 4 sets of 2 reps with 20–30 sec rest in between clusters, 3–5 min rest between sets

Squats: 5 2 Cluster: 4 sets of 2 reps with 20–30 sec rest in between clusters, 3–5 min rest between sets

Romanian Deadlifts 4 6 –

Weighted Planks 3 60 s –

**Day 2: Upper Body**

**Exercise Sets Repetition Notes**

Bench Press 5 2 Cluster: 4 sets of 2 reps with 20–30 sec rest in between clusters, 3–5 min rest between sets

Pull-ups or Pulldowns 4 6–8 –

Bent-over Rows 4 6 –

Overhead Press 3 5 –

Dumbbell Hammer Curls 3 8 –

Skull Crushers 3 8 –

**Week 9–12: Power Phase**

**Day 1: Lower Body & Core**

**Exercise Sets Repetition Notes**

Power Cleans 5 2 –

Box Squats 4 3 –

Walking Lunges 3 10 each leg

Russian Twists 3 15 –

**Day 2: Upper Body**

**Exercise Sets Repetition Notes**

Speed Bench Press 6 3 Use 50–60% of 1RM, focus on explosive movement

Weighted Pull-ups 4 5 –

Incline Bench Press 4 5 –

Face Pulls 3 12 –

Barbell Curls 3 6 –

**Week 13–16: Deload & Test Phase**

Reduce volume and intensity to allow for recovery and adaptation. Test your 1RM in squat, deadlift, and bench press at the end of week 16 to assess progress.

**S-TABLE 3:** Conversation with Google Gemini (hypertrophy) on April 30, 2024

**Inserted Question by Human:**

Please provide me with a resistance training plan to increase muscle hypertrophy over the next 16 weeks. I am a 25-year-old female and have been doing resistance training 4 times a week for the past 6 years. Previous resistance training sessions have lasted 60–90 minutes. I have access to free weights and machines, both of which I would like to use. I also have training equipment such as belts, straps, bandages that I can use, and I have a body composition scale for monitoring purposes. My body weight is 60 kg with 20% body fat. I am 165 cm tall. I would like to increase the frequency to 5 times a week. I want to increase my total muscle mass as much as possible, although I am at an advanced level. I would like to emphasise my legs and my buttocks and have a more curvy silhouette. My one-repetition maximums in the squat, hip thrust, and deadlift are 100 kg, 175 kg, and 100 kg respectively. Overall, I want to incorporate advanced training strategies such as drop sets because I enjoy them. I also want to focus on my nutrition and recovery for muscle hypertrophy. To give you an idea of how I used to train, here is my previous resistance training programme:

**Day 1 & Day 3: Lower body & core**

| Exercises | Sets | Repetitions | Intensity | Rest periods |
|---|---|---|---|---|
| Romanian Deadlifts | 4 | 8 | 1–2 reps in reserve | 3 min. |
| Squats | 3 | 10–12 | 3 reps in reserve | 90 s |
| Hip Thrusts | 3 | 8 | 1–2 reps in reserve | 120 s |
| Bulgarian Split Squats | 2 | 6–8 | 3 reps in reserve | 3 min. |
| Leg raises | 2 | 15–20 | 1–2 reps in reserve | 90 s |

**Day 2 & Day 4: Upper body**

| Exercises | Sets | Repetitions | Intensity | Rest periods |
|---|---|---|---|---|
| Rows | 4 | 8 | 3 reps in reserve | 3 min. |
| Bench press | 3 | 10 | 3 reps in reserve | 3 min. |
| Pulldown | 3 | 10–12 | 1–2 reps in reserve | 3 min. |
| Lateral raises | 2 | 15–20 | 1–2 reps in reserve | 90 s |
| Biceps curls | 3 | 15 | 1–2 reps in reserve | 90 s |
| Triceps ext. | 3 | 15 | 1–2 reps in reserve | 90 s |

**Response Generated by Google Gemini:**

16-Week Advanced Hypertrophy Training Plan (Female). This plan incorporates your advanced experience, preference for higher frequency, and desire for a curvier physique. It utilizes progressive overload and advanced techniques like drop sets. Remember, proper form and recovery are crucial. Adjust weights based on your 1RM (one-rep max).

- **Frequency: 5 days per week**
- **Training Split:**
  - Day 1: Legs & Glutes (Hypertrophy Focus)
  - Day 2: Push (Chest, Shoulders, Triceps)
  - Day 3: Rest or Active Recovery (Cardio, Yoga)
  - Day 4: Pull (Back, Biceps)
  - Day 5: Lower Body (Strength Focus)
  - Day 6 & 7: Rest
- **Warm-up (Before Each Workout):**
  - 5–10 minutes light cardio (e.g., jump rope, light jog)
  - Dynamic stretches (e.g., arm circles, leg swings)
- **Cool-down (After Each Workout):**
  - 5–10 minutes static stretches (hold each stretch for 30 seconds)
- **Nutrition and Recovery:**
  - Focus on a high-protein diet for muscle growth
  - Prioritize sleep (7–9 hours per night) for optimal recovery
  - Consider creatine for increased strength and performance

**Sample Training Program:**

**Day 1: Legs & Glutes (Hypertrophy Focus)**

**Exercise Sets Reps Rest Notes**

Squats 4 6–8 2–3 min Heavy weight, progressive overload

Leg Press (Machine) 3 10–12 90 s Moderate weight, focus on glute activation

Romanian Deadlifts 3 10 120 s Moderate weight, controlled movement

Barbell Hip Thrusts 3 10–12 120 Moderate weight, focus on Drop Set 6–8 squeezing glutes

Leg Extensions 3 12–15 60 s Lighter weight, isolate quads

Hamstring Curls 3 12–15 60 s Lighter weight, isolate hamstrings

**Day 2: Push (Chest, Shoulders, Triceps)**

**Exercise Sets Reps Rest Notes**

Incline Barbell Press 4 4–6 2–3 min Heavy weight, progressive overload

DB Bench Press 3 8–10 90 s Moderate weight, full range of motion

Overhead Press 3 8–10 90 s Moderate weight, focus on core stability

Lateral Raises 3 10–12 60 s Moderate weight, controlled movement

Triceps Pushdowns 3 12–15 60 s Moderate weight, focus on tricep extension

**Day 4: Pull (Back, Biceps)**

**Exercise Sets Reps Rest Notes**

Weighted Pull-Ups 4 AMRAP 2–3 min Focus on progressive overload (weight or reps)

Barbell Rows 3 6–8 2–3 min Heavy weight, progressive overload

Seated Cable Rows 3 10–12 90 s Moderate weight, focus on back contraction

Bicep Curls 3 10–12 60 s Moderate weight, controlled movement

Hammer Curls 3 12–15 60 s Lighter weight, isolate brachialis

**Day 5: Lower Body (Strength Focus)**

**Exercise Sets Reps Rest Notes**

Back Squats 5 5 3–4 min Very heavy weight, focus on proper form

Deadlifts 3 3 4–5 min Very heavy weight, prioritize safety and form with straps if needed

Leg Press (Machine) 3 8–10 90 s Moderate weight, maintain good

**S-TABLE 4:** Conversation with Google Gemini (strength) on April 30, 2024

**Inserted Question by Human:**

Please provide me with a resistance training plan to increase maximal strength over the next 16 weeks. I am a 25-year-old female and have been doing resistance training 4 times a week for the past 6 years. Previous resistance training sessions have lasted 60–90 minutes. I have access to free weights and machines, both of which I would like to use. I also have training equipment such as belts, straps, bandages that I can use, and I have a body composition scale for monitoring purposes. I would like to keep my training frequency at 4 times a week. I want to increase my maximal strength as much as possible, although I am at an advanced level. I want to increase my maximal strength in squat, deadlift, and bench press by 10%. My one-repetition maximums in the squat, deadlift, and bench press are 100 kg, 100 kg, and 60 kg, respectively. Overall, I want to incorporate advanced training strategies such as cluster training because I enjoy it. I also want to focus on my nutrition and recovery. To give you an idea of how I used to train, here is my previous resistance training programme:

| **Day 1 & Day 3: Lower body & core** | | | | |
|---|---|---|---|---|
| Exercises | Sets | Repetitions | Intensity | Rest periods |
| Deadlifts | 5 | 1–3 | 0–2 reps in reserve | 3–5 min. |
| Squats | 5 | 1–3 | 0–2 reps in reserve | 3–5 min. |
| Hip Thrusts | 2 | 10–12 | 3 reps in reserve | 120 s |
| Bulgarian Split Squats | 2 | 10–12 | 3 reps in reserve | 120 s |
| Leg raises | 2 | 15–20 | 1–2 reps in reserve | 90 s |
| **Day 2 & Day 4: Upper body** | | | | |
| Exercises | Sets | Repetitions | Intensity | Rest periods |
| Bench press | 5 | 1–3 | 0–2 reps in reserve | 3–5 min. |
| Rows | 4 | 8 | 1–2 reps in reserve | 3 min. |
| Pulldown | 3 | 10–12 | 3 reps in reserve | 90 s |
| Lateral raises | 2 | 15 | 1–2 reps in reserve | 60 s |
| Biceps curls | 2 | 15 | 1–2 reps in reserve | 60 s |
| Triceps ext. | 2 | 15 | 1–2 reps in reserve | 60 s |

**Response Generated by Google Gemini:**

16-Week Advanced Strength Training Plan (Female, 25 years old). This plan incorporates your experience, advanced techniques like cluster training, and focuses on maximizing strength in squat, deadlift, and bench press. It utilizes a 4-day split with a focus on progressive overload and proper recovery.

- **Important Note:**
  - This is a sample plan. Adjust weights, sets, reps, and rest periods based on your progress.
  - You mentioned a body composition scale. This can be helpful for monitoring overall progress, but prioritize strength gains through performance in the main lifts (squat, deadlift, bench press).
  - Always prioritize proper form over lifting heavier weights.
- **Frequency: 4 days per week**
- **Training Split:**
  - Day 1: Lower Body & Core (Strength Focus)
  - Day 2: Upper Body Push (Strength Focus)
  - Day 3: Rest or Active Recovery (Light cardio, yoga, mobility work)
  - Day 4: Upper Body Pull (Strength Focus)
  - Day 5, 6 & 7: Rest
- **Progression:**
  - The program utilizes progressive overload by increasing weight, reps, or sets over time. Aim to increase weight by 2.5–5 kg when you can comfortably complete all prescribed reps and sets with good form.
  - Deload week (Week 13) allows for recovery and helps prevent plateaus.

- **Advanced Technique: Cluster Training**
  - Cluster sets involve performing multiple submaximal reps with short rest periods between them. This technique helps improve technique and build strength at heavier weights.
- **Nutrition and Recovery:**
  - Focus on a balanced diet with sufficient protein intake (around 1.6–2 grams per kilogram of bodyweight) to support muscle growth and repair.
  - Prioritize sleep (7–8 hours per night) for optimal recovery.
  - Consider using a post-workout protein shake to further support muscle recovery.
- **Monitoring Progress:**
  - Retest your 1RM for squat, deadlift, and bench press every 4 weeks to track progress.
  - Monitor your bodyweight and adjust your calorie intake as needed to maintain energy levels and support strength gains.
- **Disclaimer:**
  - This is a sample plan and may need adjustments based on your individual needs and progress. It's recommended to consult a certified personal trainer for personalized guidance and to ensure proper form during exercises.
- **Warm-up (Perform before each workout):**
  - 5–10 minutes of light cardio (e.g., jump rope, jogging)
  - Dynamic stretches targeting major muscle groups involved in the workout
- **Cool-down (Perform after each workout):**
  - 5–10 minutes of static stretches targeting major muscle groups involved in the workout

**The Program (4 Days per Week):**

**Day 1: Lower Body & Core (Strength)**

**Week Exercise Sets Reps Rest Notes**

1–4 Squats 3 3 30 s 3 sets of clusters 85% of 1RM

5–8 Squats 5 1 – 90% of 1RM

9–12 Squats 3 2 – 95% of 1RM

13–16 Squats 3 5 – 75% of 1RM

1–4 Deadlift 3 3 30 s 3 sets of clusters 85% of 1RM

5–8 Deadlift 5 1 – 90% of 1RM

9–12 Deadlift 3 2 – 95% of 1RM

13–16 Deadlift 3 5 – 75% of 1RM

1–16 RDL 3 8–12 – –

1–16 Leg Press 3 8–12 – –

1–16 Glute-Ham Raise 3 8–12 – –

1–16 Anti-Rotational Core Press 3 8–12 – –

**Day 2: Upper Body Push (Strength)**

**Week Exercise Sets Reps Rest Notes**

1–4 Bench Press 3 3 30 s 3 sets of clusters 85% of 1RM

5–8 Bench Press 5 1 – 90% of 1RM

9–12 Bench Press 3 2 – 95% of 1RM

13–16 Bench Press 3 5 – 75% of 1RM

1–16 Overhead Press 3 5 – 80% of 1RM

1–16 Incline DB Press 3 8–12 – –

1–16 Dips (weighted) 3 8–12 – –

1–16 Triceps Pushdowns 3 8–12 – –

1–16 Lateral Raise 3 8–12 – –

**Day 3: Rest or Active Recovery (Light cardio, yoga, mobility work)**

**Day 4: Upper Body Pull (Strength)**

**Week Exercise Sets Reps Rest Notes**

1–4 Barbell Row 3 3 30 s 3 sets of clusters 85% of 1RM

5–8 Barbell Row 5 1 – 90% of 1RM

9–12 Barbell Row 3 2 – 95% of 1RM

13–16 Barbell Row 3 5 – 75% of 1RM

1–16 Pull-Ups – – – AMRAP

1–16 Seated Cable Row 3 8–12 – –

1–16 Face Pulls 3 8–12 – –

1–16 Bicep Curls 3 8–12 – –

**S-TABLE 5:** Conversation with GPT-3.5 (via Microsoft Copilot; hypertrophy) on April 30, 2024

**Inserted Question by Human:**

Please provide me with a resistance training plan to increase muscle hypertrophy over the next 16 weeks. I am a 25-year-old female and have been doing resistance training 4 times a week for the past 6 years. Previous resistance training sessions have lasted 60–90 minutes. I have access to free weights and machines, both of which I would like to use. I also have training equipment such as belts, straps, bandages that I can use, and I have a body composition scale for monitoring purposes. My body weight is 60 kg with 20% body fat. I am 165 cm tall. I would like to increase the frequency to 5 times a week. I want to increase my total muscle mass as much as possible, although I am at an advanced level. I would like to emphasise my legs and my buttocks and have a more curvy silhouette. My one-repetition maximums in the squat, hip thrust, and deadlift are 100 kg, 175 kg, and 100 kg respectively. Overall, I want to incorporate advanced training strategies such as drop sets because I enjoy them. I also want to focus on my nutrition and recovery for muscle hypertrophy. To give you an idea of how I used to train, here is my previous resistance training programme:

| Day 1 & Day 3: Lower body & core | | | | |
|---|---|---|---|---|
| Exercises | Sets | Repetitions | Intensity | Rest periods |
| Romanian Deadlifts | 4 | 8 | 1–2 reps in reserve | 3 min. |
| Squats | 3 | 10–12 | 3 reps in reserve | 90 s |
| Hip Thrusts | 3 | 8 | 1–2 reps in reserve | 120 s |
| Bulgarian Split Squats | 2 | 6–8 | 3 reps in reserve | 3 min. |
| Leg raises | 2 | 15–20 | 1–2 reps in reserve | 90 s |
| **Day 2 & Day 4: Upper body** | | | | |
| Exercises | Sets | Repetitions | Intensity | Rest periods |
| Rows | 4 | 8 | 3 reps in reserve | 3 min. |
| Bench press | 3 | 10 | 3 reps in reserve | 3 min. |
| Pulldown | 3 | 10–12 | 1–2 reps in reserve | 3 min. |
| Lateral raises | 2 | 15–20 | 1–2 reps in reserve | 90 s |
| Biceps curls | 3 | 15 | 1–2 reps in reserve | 90 s |
| Triceps ext. | 3 | 15 | 1–2 reps in reserve | 90 s |

**Response Generated by Microsoft Copilot:**

Certainly! Let's create a customized 16-week resistance training plan to maximize muscle hypertrophy based on your goals and current level. Since you're already at an advanced level, we'll incorporate some advanced strategies while emphasizing your legs, buttocks, and overall physique. Here's a comprehensive plan for you:

- **Frequency: 5 days per week**
- **Training Split:**
  - Day 1: Lower Body & Core
  - Day 2: Upper Body
  - Day 3: Rest or Active Recovery
  - Day 4: Lower Body & Core
  - Day 5: Upper Body
  - Day 6–7: Rest or Active Recovery
- **Exercise Selection:**
  - I'll include compound movements that target multiple muscle groups and isolation exercises to focus on specific areas.
  - You have access to free weights and machines, so we'll utilize both.
- **Reps and Sets:**
  - Aim for 10 sets per muscle group per week for hypertrophy.
  - Best results come from 4–5 sets of 8–12 reps per muscle group each workout.
  - Alternate training each muscle group at least twice throughout the week, and we'll add a 5$^{th}$ day for specific isolation work.

– **Nutrition and Recovery:**
  - Prioritize adequate protein intake (around 1.2–2.0 grams per kilogram of body weight) to support muscle growth.
  - Ensure you're getting enough **quality sleep** for optimal recovery.
– **Advanced Strategies:**
  - Drop Sets: Incorporate drop sets occasionally to challenge your muscles further. After completing a set, immediately reduce the weight and perform additional reps.

Remember to track your progress, adjust weights as needed, and listen to your body. Consistency and progressive overload are key for hypertrophy. Enjoy your workouts, and let's build that curvy silhouette!

**Sample Workout Plan:**

**Day 1 & 4: Lower Body & Core**

**Exercise Sets Reps Rest Notes**

RDL 4 8 2–3 min

Squats 3 10–12 2–3 min

Hip Thrusts 3 8 2–3 min

Bulgarian Split Squats 2 6–8 1–2 min each leg

Leg Raises 2 15–20 1–2 min

**Day 2 & 5: Upper Body**

**Exercise Sets Reps Rest Notes**

Barbell Rows 4 8 2–3 min –

Bench Press 3 10 2–3 min –

Lat Pulldowns 3 10–12 1–2 min –

Lateral Raises 2 15–20 1–2 min –

Biceps Curls 3 15 1–2 min –

Triceps Extensions 3 15 1–2 min –

**Day 3: Rest or Active Recovery (Light Cardio, Yoga, etc.)**

**S-TABLE 6:** Conversation with GPT-3.5 (via Microsoft Copilot; strength) on April 30, 2024

**Inserted Question by Human:**

Please provide me with a resistance training plan to increase maximal strength over the next 16 weeks. I am a 25-year-old female and have been doing resistance training 4 times a week for the past 6 years. Previous resistance training sessions have lasted 60–90 minutes. I have access to free weights and machines, both of which I would like to use. I also have training equipment such as belts, straps, bandages that I can use, and I have a body composition scale for monitoring purposes. I would like to keep my training frequency at 4 times a week. I want to increase my maximal strength as much as possible, although I am at an advanced level. I want to increase my maximal strength in squat, deadlift, and bench press by 10%. My one-repetition maximums in the squat, deadlift, and bench press are 100 kg, 100 kg, and 60 kg, respectively. Overall, I want to incorporate advanced training strategies such as cluster training because I enjoy it. I also want to focus on my nutrition and recovery. To give you an idea of how I used to train, here is my previous resistance training programme:

| Day 1 & Day 3: Lower body & core | | | | |
|---|---|---|---|---|
| Exercises | Sets | Repetitions | Intensity | Rest periods |
| Deadlifts | 5 | 1–3 | 0–2 reps in reserve | 3–5 min. |
| Squats | 5 | 1–3 | 0–2 reps in reserve | 3–5 min. |
| Hip Thrusts | 2 | 10–12 | 3 reps in reserve | 120 s |
| Bulgarian Split Squats | 2 | 10–12 | 3 reps in reserve | 120 s |
| Leg raises | 2 | 15–20 | 1–2 reps in reserve | 90 s |
| **Day 2 & Day 4: Upper body** | | | | |
| Exercises | Sets | Repetitions | Intensity | Rest periods |
| Bench press | 5 | 1–3 | 0–2 reps in reserve | 3–5 min. |
| Rows | 4 | 8 | 1–2 reps in reserve | 3 min. |
| Pulldown | 3 | 10–12 | 3 reps in reserve | 90 s |
| Lateral raises | 2 | 15 | 1–2 reps in reserve | 60 s |
| Biceps curls | 2 | 15 | 1–2 reps in reserve | 60 s |
| Triceps ext. | 2 | 15 | 1–2 reps in reserve | 60 s |

**Response Generated by Microsoft Copilot:**

Certainly! To enhance your maximal strength over the next 16 weeks, we'll focus on a well-structured resistance training plan. Given your advanced level and desire to increase strength in squat, deadlift, and bench press, here's a customized program:

- **Frequency: 4 days per week (with 3 rest days)**
- **Training Split:**
  - Day 1: Lower Body & Core
  - Day 2: Upper Body
  - Day 3: Rest or Active Recovery
  - Day 4: Lower Body & Core
  - Day 5: Upper Body
  - Day 6–7: Rest or Active Recovery
- **Nutrition and Recovery:**
  - Prioritize protein intake for muscle repair and growth.
  - Get adequate sleep (7–9 hours) to support recovery.
  - Hydrate well and manage stress.
- **Advanced Strategies:**
  - Cluster Training: Since you enjoy it, incorporate cluster sets. These involve breaking a set into smaller clusters with short rest intervals (e.g., 3 sets of 2 reps with 20 seconds rest between reps).
  - Progressive Overload: Continuously increase the weight or resistance to challenge your muscles. This is crucial for strength gains[1].
  - Neural Drive Enhancement: Focus on explosive lifts and fast concentric movements to engage high-threshold motor units and improve neural drive.

- Visualization: Spend 15–20 minutes daily visualizing successful lifts. Make it vivid and sensory-rich.

Remember, consistency and gradual progression are key. Monitor your progress using your body composition scale and adjust as needed. Good luck with your strength journey!

**Sample Workout Plan:**

**Day 1 & 3: Lower Body & Core**

**Exercise Sets Reps Rest Notes**

Deadlifts 5 1–3 3–5 min 0–2 RIR

Squats 5 1–3 3–5 min 0–2 RIR

Hip Thrusts 2 10–12 120 s 3 RIR

Bulgarian Split Squats 2 10–12 120 s 3 RIR

Leg Raises 2 15–20 90 s 1–2 RIR

**Day 2 & 4: Upper Body**

**Exercise Sets Reps Rest Notes**

Bench Press 5 1–3 3–5 min 0–2 RIR

Rows 4 8 3 min 1–2 RIR

Pulldown 3 10–12 90 s 3 RIR

Lateral Raises 2 15 60 s 1–2 RIR

Biceps Curls 2 15 60 s 1–2 RIR

Triceps Extensions 2 15 60 s 1–2 RIR

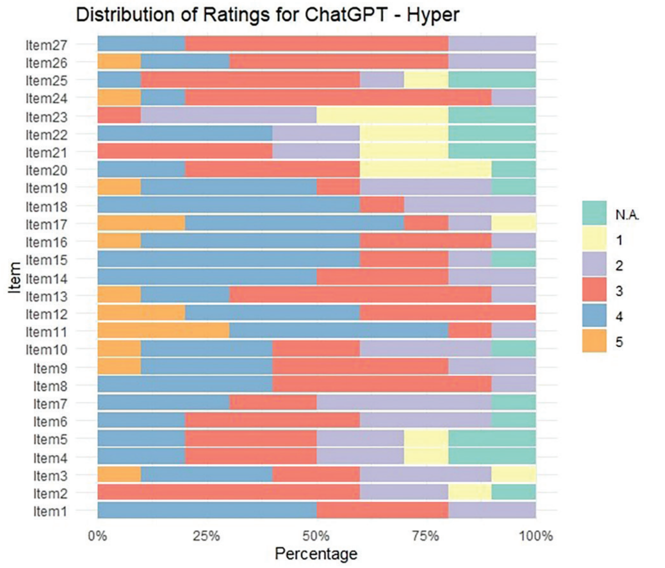


S-FIGURE 1. Likert Scale Chart of GPT-3.5 (via ChatGPT; hypertrophy)

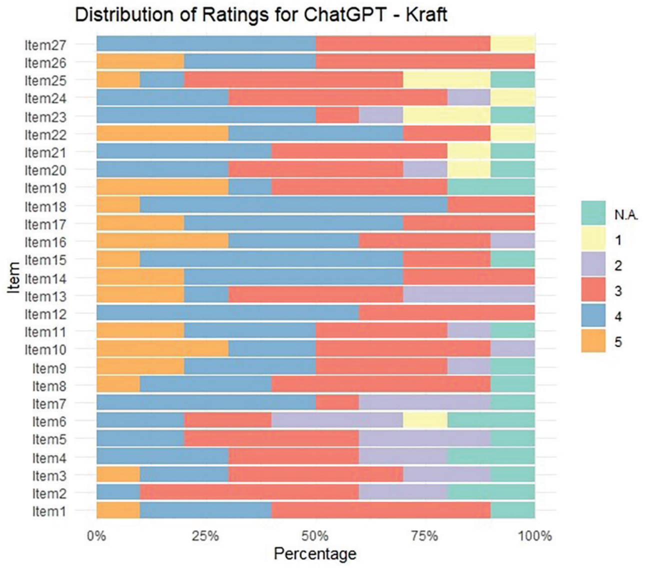


**S-FIGURE 2.** Likert Scale Chart of GPT-3.5 (via ChatGPT; strength)

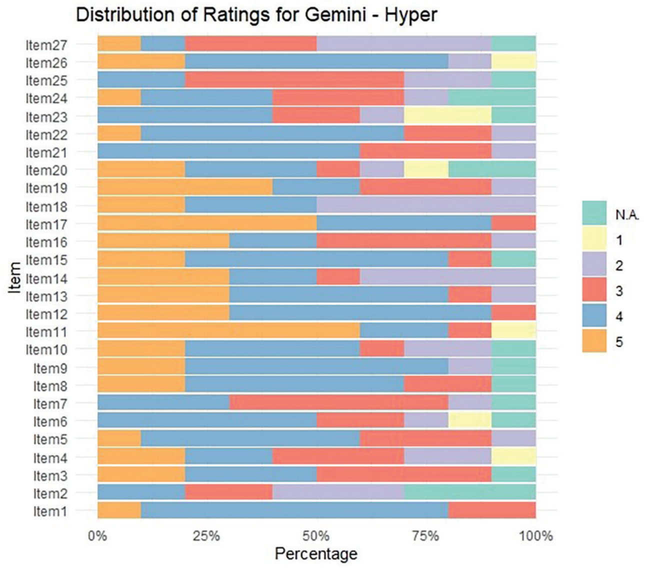


**S-FIGURE 3.** Likert Scale Chart of Google Gemini (hypertrophy)

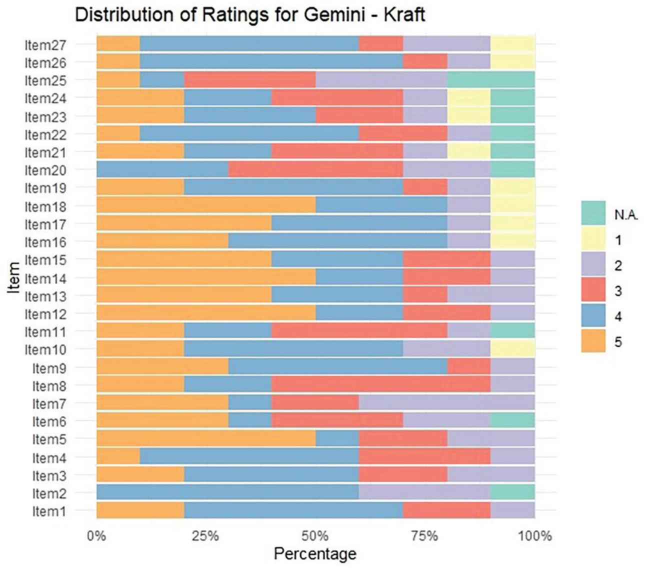


**S-FIGURE 4.** Likert Scale Chart of Google Gemini (strength)

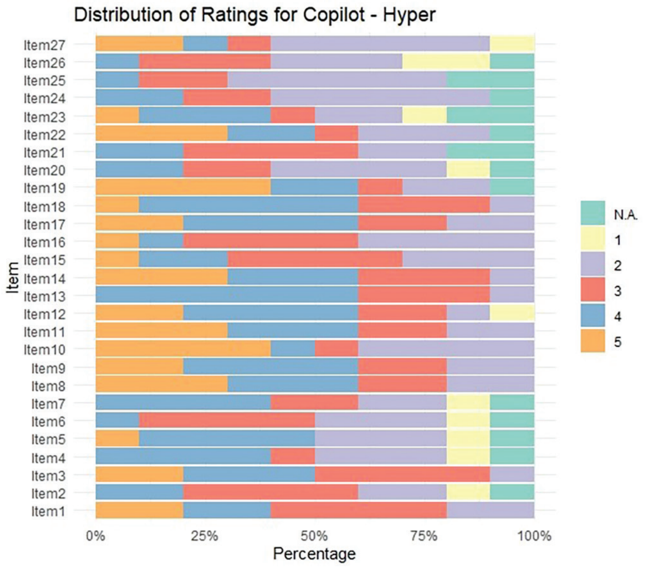


**S-FIGURE 5.** Likert Scale Chart of GPT-3.5 (via Microsoft Copilot; hypertrophy)

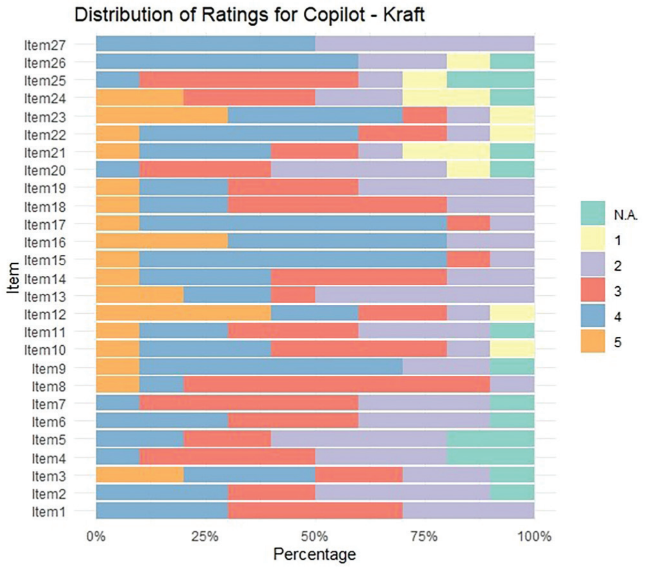


**S-FIGURE 6.** Likert Scale Chart of GPT-3.5 (via Microsoft Copilot; strength**)**

Articles published in the Biology of Sport are licensed under an open access Creative Commons CC BY 4.0 license.
